# Astrocyte-neuron lactate shuttle plays a pivotal role in sensory-based neuroprotection in a rat model of permanent middle cerebral artery occlusion

**DOI:** 10.1038/s41598-023-39574-9

**Published:** 2023-08-07

**Authors:** Mehwish S. Bhatti, Ron D. Frostig

**Affiliations:** 1 Department of Neurobiology and Behavior, School of Biological Sciences, University of California, Irvine, Irvine, CA USA; 2 Department of Biomedical Engineering, School of Engineering, University of California, Irvine, Irvine, CA USA; 3 Center for Neurobiology of Learning and Memory, University of California, Irvine, Irvine, CA USA

**Keywords:** Neuroscience, Health care, Medical research

## Abstract

We have previously demonstrated protection from impending cortical ischemic stroke is achievable by sensory stimulation of the ischemic area in an adult rat model of permanent middle cerebral artery occlusion (pMCAo). We have further demonstrated that a major underpinning mechanism that is necessary for such protection is the system of collaterals among cerebral arteries that results in reperfusion of the MCA ischemic territory. However, since such collateral flow is weak, it may be necessary but not sufficient for protection and therefore we sought other complementary mechanisms that contribute to sensory-based protection. We hypothesized that astrocytes-neuron lactate shuttle (ANLS) activation could be another potential underpinning mechanism that complements collateral flow in the protection process. Supporting our hypothesis, using functional imaging, pharmacological treatments, and postmortem histology, we showed that ANLS played a pivotal role in sensory stimulation-based protection of cortex and therefore serves as the other supporting mechanism underpinning the protection process.

## Introduction

**Figure 1:**
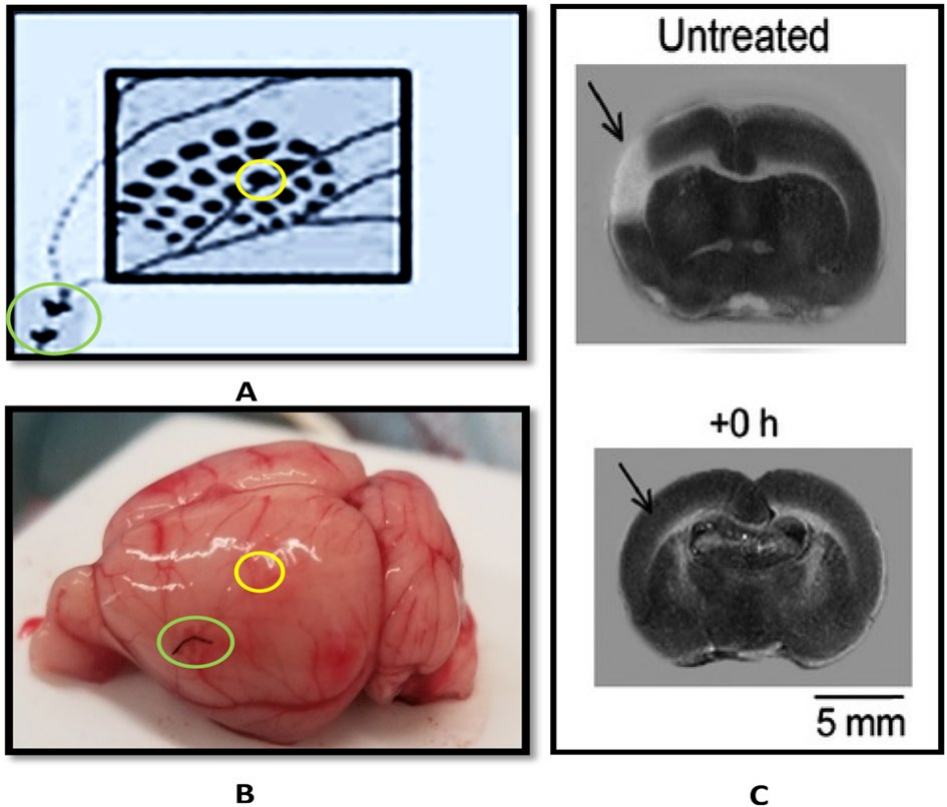
Location of (middle cerebral artery) MCA, barrel fields and infarct. (**A**) shows the MCA occlusion location (green bounding circle) where it is tied and the barrel cortex (in yellow, C2 whisker-barrel), image taken from our previous study^1^. (**B**) The green ellipse shows the black thread used for MCA-occlusion and the yellow circle shows approximate location of C2-whisker response. (**C**) Top: TTC slice of the infarct region in white (pointed by an arrow) in a permanent middle cerebral artery occlusion (pMCAo) rat with no sensory stimulation; Bottom: TTC slice of the protected region (no white region, pointed by an arrow) in a pMCAo rat with immediate (+ 0 h) stimulation (2 h-long C2 whisker stimulations delivered right after pMCAo), image taken from our previous study^5^.

Using permanent middle cerebral artery occlusion (pMCAo) in a rat model of ischemic stroke (Fig. 1) we have demonstrated protection from impending ischemic stroke by sensory stimulation delivered within a two-hour time window of protection after ischemic onset^1–8^. What are the mechanisms underpinning this sensory-based neuroprotection in this rat model? We have demonstrated that a necessary component for such protection is the pial collateral system (leptomeningeal anastomoses) that provide retrograde blood flow to the occluded MCA from other cortical arteries resulting in reperfusion of the ischemic area^1^. Applying doppler optical coherence tomography (DOCT) that enables direct quantification of cortical blood flow and flux, we found that sensory stimulation following pMCAo indeed enhanced retrograde collateral blood flow and flux into the permanently occluded MCA^9^. However, despite such enhancement, the collateral blood flow and flux remained at a very low level during the initial critical hours for protection following pMCAo^9^. These findings suggested that, although collateral flow is necessary for neuroprotection of the ischemic cortical tissue, it may not be sufficient. We therefore searched for another complementary mechanism that could also participate in sensory-based neuroprotection.

Brain energy management is based on collaborative and dynamic interactions of neuron-glia-vasculature (NGV) involved in energy production, transfer, and utilization^10–13^. In recent years there is growing supporting evidence for the importance of the astrocyte-neuron lactate shuttle (ANLS) that provides lactate as a source for ‘on demand’ energy needs of activated neurons and preferential energy source for some neurons^10,11,14–21^. There are two forms of ANLS that occur in healthy animal models that result in lactate release from astrocytes^15^. One is based on astrocytic glycogenolysis and the other is based on astrocyte's glucose uptake that activates its metabolism through aerobic glycolysis, both resulting in lactate production^21,22^. A recent study has established that in healthy animals energy supply to neurons critically depends on the level of neuronal activity^15^. At low levels of neuronal activity neurons uptake glucose directly from the blood, whereas high levels or intense neuronal activity result in a shift from glucose to lactate use by neurons. Since sensory-based protection following pMCAo depends on evoked activation of neurons in the ischemic area, we hypothesized that similar neuronal activity-dependent mechanisms are involved in the ischemic cortex. Accordingly, ANLS could be activated in response to sensory-evoked neuronal stimulation, thereby aiding neuroprotection of the ischemic territory. To address this hypothesis, following pMCAo, we used pharmacological manipulations that blocked the lactate transporters on cortical neurons located within the ischemic territory during sensory stimulation. Our results support the hypothesis that the lactate shuttle is also a necessary complementary underpinning mechanism for sensory-based neuroprotection in the rat model of ischemic stroke and highlights the importance of sensory-activated astrocytes in neuroprotection. These findings, together with our previous findings that show how sensory stimulation minimizes the post-pMCAo build-up of infarct-predicting widespread cortical synchrony of spontaneous local field potentials (LFPs)^23,24^, demonstrate how protection from impending ischemic stroke by sensory stimulation is multidimensional involving all components of NGV from the collateral system to ANLS and neuronal activity.

## Materials and methods

All procedures followed NIH guidelines and were approved by UC Irvine Animal Care and Use Committee (protocol #: AUP-21-065). All methods are reported in accordance with the ARRIVE guidelines.

### Animals

Thirty-two experimental subjects, 230–450 g male Sprague Dawley rats (Charles River Laboratories, Wilmington, MA, USA) were individually housed in enriched cages and placed in a temperature, humidity and light-controlled room following a twelve-hour-cycle (6am-6 pm). Each rat was handled daily for twenty minutes for 3–5 days, prior to the experiment.

### Reagents

The lactate shuttle between astrocytes and neurons is known to act through monocarboxylate transporters (MCT). MCTs are ubiquitously expressed plasma membrane proteins responsible for proton-linked transfer of molecules with one carboxylate group, across the cell membrane^25^. The transporters are expressed by both astrocytes and neurons. In the brain, the endothelial and glial cells express MCT1 and MCT4, whereas neurons express MCT2. MCTs 1 and 4 are presumed to primarily release lactate whereas; MCT2 takes up lactate^26^. α-Cyano-4-hydroxycinnamate (4-Cin) is a competitive, non-transportable inhibitor of MCTs. Previous reports have shown that 4-Cin selectively inhibits MCT2 rather than MCTs 1 and 4 due to the difference in IC_50_ values among the MCT isoforms^27–29^. Based on extensive literature review of 4-Cin concentrations, 0.5 ml of 8.6 mM 4-Cin dissolved in 4.5% dimethyl sulfoxide (Dmso, a polar solvent) is used in this study^28^.2,3,5-triphenyltetrazolium chloride (TTC) is used for post-mortem histology to highlight and quantify the infract area. All drugs are purchased from Sigma Aldrich, Saint louis, MO, USA.

### Experimental design

**Figure 2:**
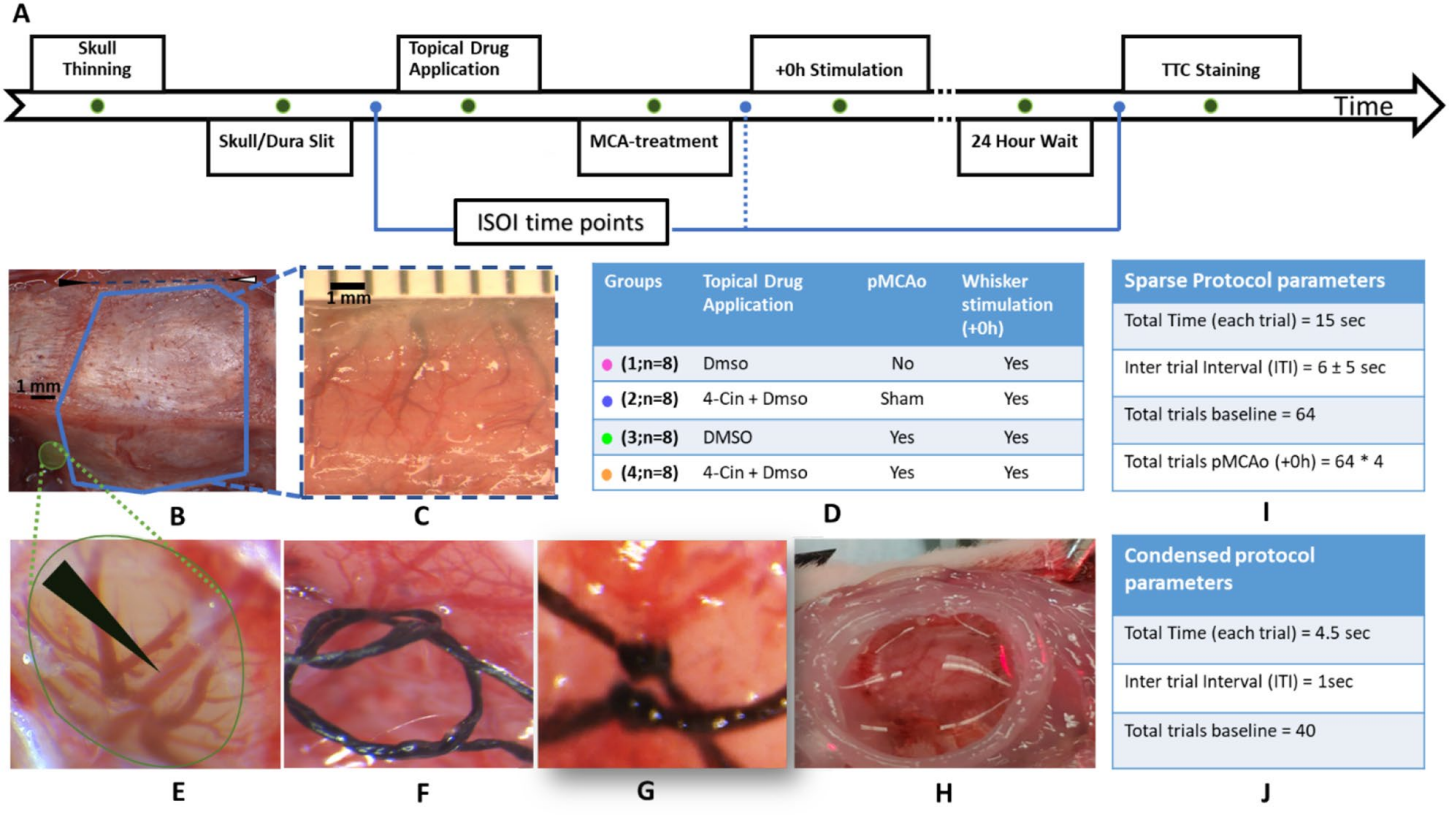
Experimental design and surgical procedures. (**A**) The detailed timeline of the experiment. (**B**) Black and white arrowhead (top) show bregma and lambda respectively. The thick blue line shows the skull area thinned for ISOI. Small green area is thinned for pMCAo. (**C**) Thinned area (approximate thickness range 24–32 um) prepared for intrinsic signal optical imaging, ISOI; underlying cortex and vessels are clearly visible. (**D**) The drug intervention, MCA-treatment, details of whisker stimulation and the assigned color for each experiment group are tabulated. (**E**) MCA after craniotomy and durotomy. Black arrowhead points to dorsal MCA. (**F**) Two threads are passed under MCA and loosely knotted (**G**) Permanent double knotted MCA. (**H**) A petroleum jelly (Vaseline) well filled with saline/drug. (**I**,**J**) tabulates parameters of sparse and condensed whisker stimulation protocols, respectively.

In this study, we applied a within subject design where a baseline is established and compared to post manipulation result. Thirty-two subjects were randomly assigned to one of four experimental groups by an experimenter blind to the experiment protocols. Immediate post occlusion whisker stimulation is referred to as + 0 h. All the experimental groups are shown in detail in Fig. 2D. Rats in group 1 (n = 8) received application of vehicle (Dmso, 4.5%) and + 0 h whisker stimulation but no pMCAo. Rats in group 2 (n = 8) received sham treatment for MCA occlusion, MCT-inhibitor (4-CIN, 8.6 mM) and + 0 h stimulation. Rats in group 3 (n = 8) received pMCAo, vehicle (Dmso) and + 0 h stimulation. Rats in group 4 (n = 8) received pMCAo, MCT inhibitor (4-Cin), and immediate post occlusion whisker stimulation (+ 0 h). Groups 1–3 serve as three different control groups to group 4. The detailed timeline of the complete experimental protocol is shown in Fig. 2A.

### Surgical preparation

At the beginning of each experiment, subjects were briefly anesthetized with 4% Isoflurane and injected intraperitoneally (ip) with sodium pentobarbital bolus (55 mg/kg body weight (bw)). Supplemental injections (14 mg/kg bw) were given as necessary to maintain loss of withdrawal reflex to toe/tail pinch. C2 whisker was identified, and the remaining whiskers were cut before the start of surgical procedures. 5% dextrose (3 mL) and atropine (0.05 mg/kg, bw) were administered at the beginning and end of day 1 of the experiment. Body temperature was continuously measured via a rectal probe and maintained at 37° Celsius by a self-regulating thermal blanket. Heart rate and partial oxygen saturation were also monitored throughout the experiment.

A midline incision was made, and soft tissue was resected to expose a ~ 7 mm × 7 mm ‘imaging’ area of the skull over the left primary somatosensory cortex (rostro medial corner positioned caudal and lateral from bregma) was thinned to ~ 24–32 μm using a dental drill as shown in Fig. 2B,C. After baseline intrinsic signal optical imaging (ISOI) of whisker C2 whisker functional representation (WFR), skull was lifted and 1–4 slits in dura were made at or around the area C2 WFR (see below).

At the end of the imaging sessions on day 1, analgesic (Flunixin meglumine) was injected subcutaneously (2 mg/kg). The closed wound was covered with topical antibiotics (Antimax, Petco), and rats were monitored while recovering from anesthesia. Rats were returned to their home cage and allowed to recover overnight prior to 24 h (referred to as 24 h) ISOI. Following the 24 h ISOI, rats were euthanized, with lethal dose of Euthasol (2 ml, ip) and prepared for histology. The experiment timeline and procedures are shown in Fig. 2.

### Skull-dura slits

**Figure 3:**
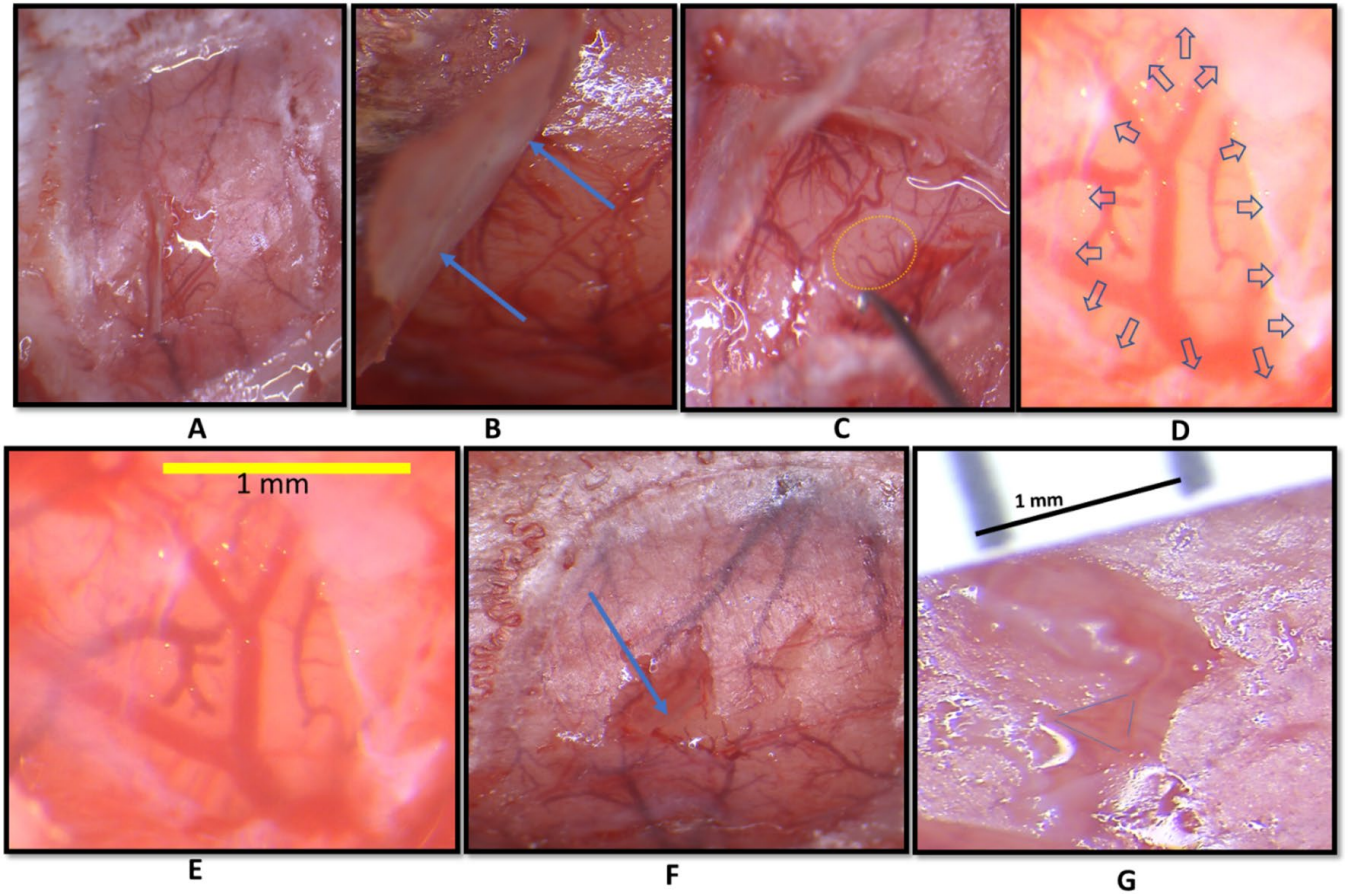
Aligned skull and dura slits. (**A**) A small opening is made in the thinned skull to lift part of it. The white streak is the reflection of overhead light. (**B**) Blue arrows show lifted skull. (**C**) A small opening is made in dura with a 30G bent needle hook. (**D**,**E**) dura slit of desired size is made. Blue arrows show retraced dura after the slit at 120X magnification. (**F**) The skull-slit halfway toward its original position, blue arrow. (**G**) Aligned dura-skull slits at 150X magnification bounded by blue dotted triangle.

The procedure of dura slits was adapted and improved from our previously reported procedure^30^. In this procedure full craniotomy of the imaging window was replaced by small, aligned skull slits with dura slits. All steps were performed at 50–120 × magnification and shown in Fig. 3. A small opening was made in the thinned skull with fine forceps and the skull flap was gently lifted and bent as shown in Fig. 3A,B. Notably, the skull is only lifted but not removed or separated. The tip of 30G needle was bent to grab and lift the dura up, away from the cortex as shown in Fig. 3C. The slit of desired size was made in the dura with the sharp part of the needle while it was lifted and made no contact with the cortex (Fig. 3D). After the slit was cut to the right size the needle was carefully separated from the dura. Using micro scissors, a small slit in the skull was made, aligned to the dura slit(s). The thin skull was then returned to its original position as shown in Fig. 3G. Zoomed in images of dura slit and aligned dura-skull slit are shown in Fig. 3F,G, respectively. By making aligned slits in both thinned skull and dura, this procedure ensures minimal tissue damage, prevents cortical herniation, and reduces cortical movement artifacts during imaging.

### Permanent dorsal middle cerebral artery occlusion (pMCAo)

This procedure was first demonstrated and described by Davis et al.^31^. Briefly, the base of the left middle cerebral artery is permanently occluded at the M1 segment blocking flow to all MCA cortical branches. To do this, the skull and dura are carefully removed from a 1.5 × 1.5 mm ‘surgical window’ just anterior and lateral to the imaging window (over the M1 segment of MCA, just distal to MCA’s lenticulostriate branches and proximal to any cortical branching) as shown in Fig. 2B,E. A small needle is threaded with 8–0 silk thread and passed at two locations through the pial layer of the meninges, below MCA as shown in Fig. 2F. Both threads are then tightly tied around MCA (Fig. 2G). Care is taken to avoid damaging the artery, and experiments are terminated if MCA is hemorrhaged. For the sham surgery group, the same procedure is used but without the final tying. The location of knotted MCA with respect to the barrel fields and the approximate location of C2 barrel is shown in Fig. 1A,B.

### Drug administration

A petroleum jelly (Vaseline) well was made around the imaging window and filled with saline for baseline ISOI as shown in Fig. 2H. The well was then filled with the drug/vehicle and is covered with a cover slip. The drug was allowed to diffuse to the cortical tissue through skull/dura slits, for one hour before pMCAo and for two hours after pMCAo as two hours after pMCAo is the critical time window for sensory-based neuro-protection^6^.

### Intrinsic signal optical imaging (ISOI)

**Figure 4:**
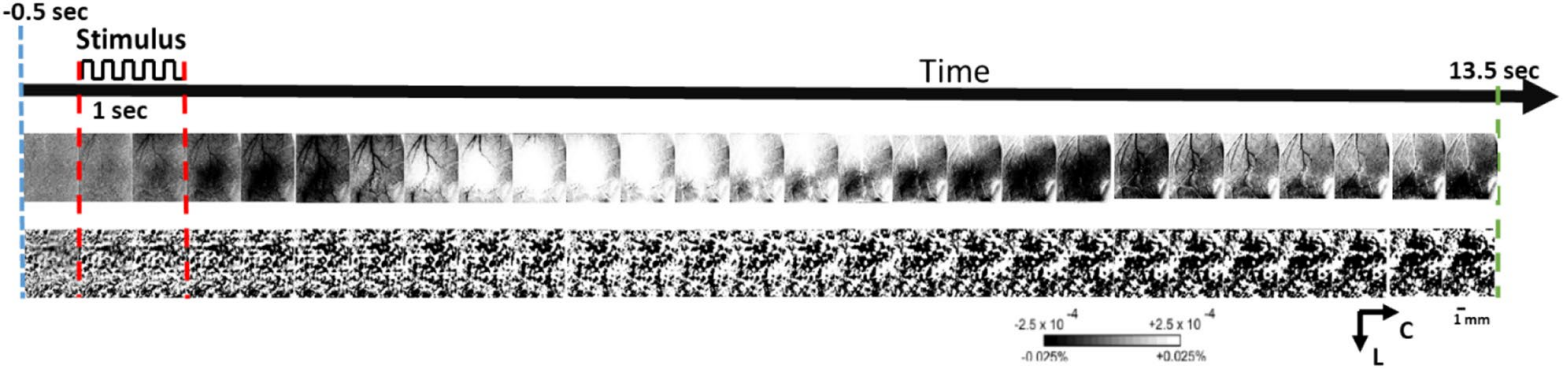
ISOI-WFR of whisker C2 during sparse stimulation protocol. Blue line shows the start of 500 ms prestimulus image. Red lines show the start and end of 5 Hz-1s-long whisker stimulation. The top row shows a typical ISOI-WFR 3-phase response to stimulation during baseline, whereas the bottom row shows the absence of the ISOI-WFR in response to the identical stimulation in the presence of MCT inhibitor. The two series of images are summed responses of 64 trials. Linear gray scale bar indicates intrinsic signal strength (± 0.00025), C and L denotes caudal and lateral respectively. Each frame is ~ 7 mm × 7 mm. Black and white streaks are large surface blood vessels artifacts.

A detailed description of ISOI data acquisition and analysis can be found elsewhere^32–34^. Briefly, a charge coupled device (CCD) camera (16-bit Cascade 512F, Photometrics, Tucson, AZ, USA) equipped with an inverted 50 mm AF Nikon lens (1:1:8, Melville, NY, USA) combined with an extender (model PK-13, Nikon, Melville, NY, USA) is used for imaging and controlled by V++ Precision Digital Imaging System software (Digital Optics, Auckland, NZ). Data is acquired in 100 ms frames that are summed to 500 ms frames to improve signal-to-noise ratio. The cortex is illuminated with a single red-light emitting diode (635 ± 15 nm wavelength). Unless otherwise noted (e.g., Fig. 4), ISOI was applied at pre-pMCAo baseline, and at 24 h post pMCAo, therefore the second imaging time point in Fig. 1 is only represented by a broken line.

### Imaging/whisking protocols

Two types of whisker stimulation protocols, *sparse* and *condensed*, are applied during imaging at baseline and at 24 h. Sparse protocol is used as it produces the same temporal sequence of functional response phases as also imaged by powerful (high-Tesla) BOLD-fMRI, a protocol that was consistently applied in all our previous work^1–8^. A condensed protocol is used because it mimics the naturalistic pattern of whisking in awake animals^35^.

*Sparse protocol:* During each 15 s trial of sparse protocol, 1.5 s of pre-stimulus data, 1 s of during stimulus and 13.5 s of post-stimulus data was collected, with a 6 ± 5 s random inter-trial interval.

*Condensed protocol*: During each 4.5 s trial of condensed protocol, 1.5 s of pre-stimulus data, 1 s of during stimulus and 2 s of post-stimulus data was collected, with a 1 s constant inter-trial interval.

For both protocols, stimulus consisted of a single whisker being deflected by 9° in the rostral-caudal direction at a rate of 5 Hz for a total stimulus duration of 1 s in each trial. Data is collected in blocks of 64 stimulation trials for sparse and in blocks of 40 for condensed whisking protocol. All post pMCAo (+ 0 h stimulations) consisted of four blocks of 64 sparse whisking stimulations which have previously been reported as neuroprotective in this model (for reference Fig. 1C)^1–8^. The location of infarct after pMCAo in rats that do not receive whisker stimulation is shown in Fig. 1C. In all animals, 256 trials of whisker stimulation immediately after pMCAo are referred as + 0 h stimulation^1,2^. The parameters of both sparse and condensed protocols are shown in Fig. 2I-J, respectively.

### Imaging analysis

**Figure 5:**
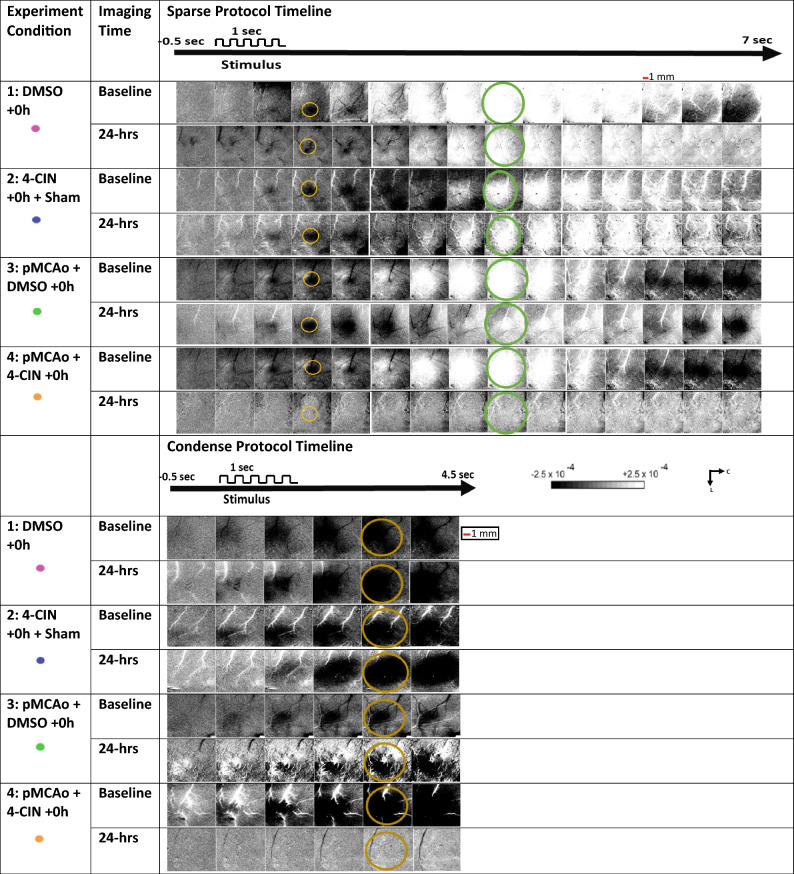
A representative result for each experimental group following the sparse whisker stimulation protocol (upper half) and condensed whisker stimulation (lower half). The ISOI results at baseline and at 24 h are shown for each experimental group (description provided under ‘Experiment condition’ column). The 1 s time mark denotes the start and end of 1 s 5 Hz-whisker stimulation pulse train. The yellow and green circles show the specific frames which include the regions of interest used for quantification of initial dip and overshoot, respectively. Linear gray scale bar indicates intrinsic signal strength ± 0.00025, C and L denotes caudal and lateral respectively. Each frame is ~ 7 mm × 7 mm. Black and white streaks are large surface blood vessels artifacts. Thick red line is scale for 1 mm.

From raw images, ratio images were created from calculating fractional change (FC) values by dividing each 500 ms frame of post-stimulus signal activity by the 500 ms frame of pre-stimulus intrinsic signal activity collected immediately before stimulus onset. WFR for sparse protocol shows three distinct phases following stimulation. The three phases are, in the order of their appearance, the initial dip (typically dark in relation to pre-stimulus baseline), the overshoot (typically bright in relation to baseline) and the undershoot (topically dark in relation to baseline)^34,36^ see for example the top row Fig. 4. When the cortex is illuminated by red (635 nm), the initial dip represents the increase in deoxyhemoglobin due to immediate oxygen support to neuronal activation, the overshoot represents the vascular response of blood flow into the activated area due to increase in oxyhemoglobin (similar to the BOLD response of fMRI), and the underpinning source of the undershoot is still unclear^36,37^. As in our previous publications, we have analyzed only the first two phases and therefore Fig. 5 shows only 7 s of data acquisition rather than 13.5 as shown in Fig. 4. The condensed protocol, however, only shows a single phase of growing initial dip.

### Spatial analysis

The ratio images containing the maximum areal extent for each intrinsic signal phase were selected and Gaussian filtered (half width = 5). The areal extent was quantified at a threshold level of 1.75 × 10^−4^ for initial dip and at 2.5 × 10^−4^ (fractional change ΔR/R units) threshold for the overshoot, away from zero. Peak amplitude was quantified in fractional change units for the pixel with peak activity within the areal extent. Notably, for comparing changes between baseline and 24 h in all experimental groups, the pixel of peak intensity was selected within the area of the skull-dura slit (drug diffusion area).

### Temporal analysis

As spatial parameters for initial dip and overshoot are based on single 500 ms frame, temporal profiles were acquired from all the 500 ms frames of a single selected peak value pixel within the area of the aligned-skull-dura slits. The timing parameters had previously been reported for the WFR and temporal profile of perfusion are now used for ISOI-WFR analysis^34,38^. The detailed analysis and results of all timings parameters are reported in supplementary materials (Figure S1 & Figure S2).

### Histology (staining for infarct)

The brain was sectioned into 2 mm coronal slices, and incubated in 2% TTC solution at 37 °C for 20 min in the dark^39^. TTC is enzymatically reduced, producing formazan (a bright red byproduct), by dehydrogenases in active mitochondria. Red stain intensity correlates with the number and functional activity of mitochondria, unstained (white) areas are indicative of infarct^40^.

### TTC analysis

The TTC-stained sections were photographed with a digital camera, and images were analyzed using ImageJ software (National Institute of Health). The total infarct volume was determined by multiplying the infarct area of each slice by the thickness of that slice and final volumes were then corrected for edema. An observer blind to the experimental groups performed the volume calculation. Small damage at the pMCAo surgical site was readily distinguished from the large ischemic infarct and was excluded from infarct analysis. The images with infarct in group 4, were superimposed on the images obtained from the rat brain atlas (Pixanos and Watson)^41^. The atlas images were selected based on landmarks observed in the slices.

### Statistical analysis

For ISOI-WFR imaging data, repeated measures analysis of variance (RM-ANOVA) was run to analyze potential differences between baseline and 24 h after pMCAo in all experimental groups. Repeated measures were performed for one between subject’s variable (experiment groups, 1–4) and one within subjects (time, baseline vs 24 h) followed by post hoc contrasts to identify which groups differed from baseline at 24-h. Alpha level was set to 0.05, and Bonferroni corrections were applied to account for multiple contrasts. Infarct volume comparisons were performed by employing two-sample t-tests. One-way ANOVA was performed with post hoc Bonferroni corrections to ensure that no statistical difference exists between baseline values of all groups (1–4). All statistics and plotting were performed using PRISM (GraphPad version 9). Results are expressed as means and standard errors.

## Results

### Blocking ANLS leads to complete elimination of spatial response of whisker functional representation (WFR) during treatment time

Blocking ANLS through MCTs affects the WFR during treatment time, an effect imaged during MCT inhibitors application in groups 2 and 4. Figure 4 shows a representative example of the ISOI-WFR in the absence (top row) and presence (bottom row) of the MCT inhibitor where the distinct three phases of the WFR disappear. This effect was present only during MCT inhibition through 4-Cin application (groups 2,4).

**Figure 6:**
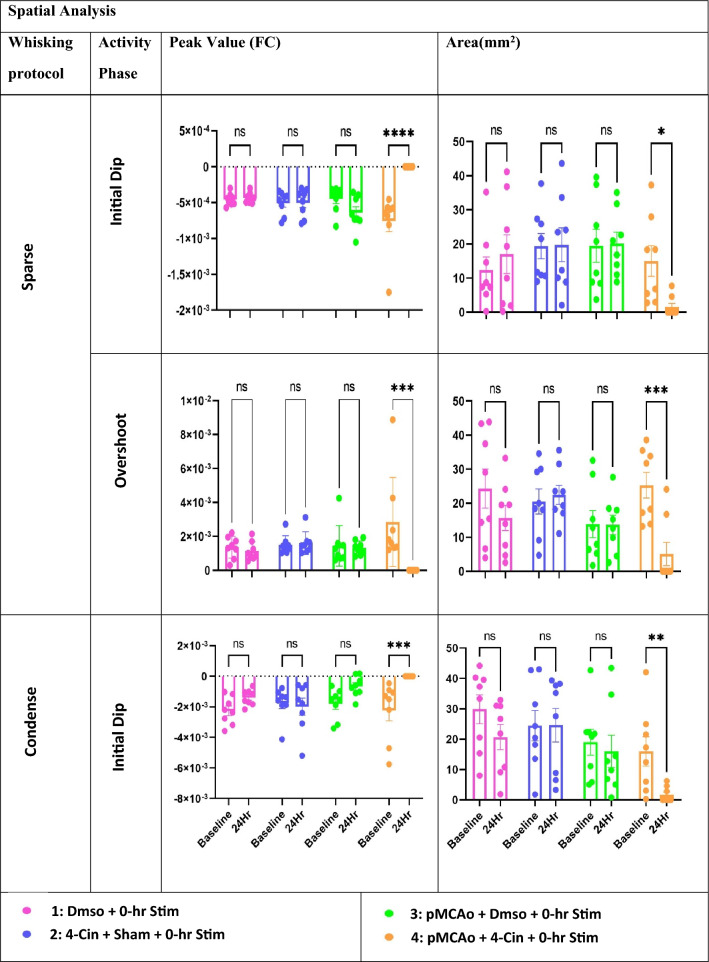
The spatial quantification of ISOI-WFR phases for the two stimulation (sparse & condensed) paradigms. Top row: peak amplitude and area of the initial dip following application of the sparse whisker stimulation at baseline vs. 24 h(24Hr) for each of the experimental groups. Graphs show a significant difference only for group 4 at baseline vs. 24 h for both peak and area (****p < 0.0001 and *p < 0.05). Middle row: peak amplitude and area of the overshoot at baseline vs. 24 h following sparse protocol is shown for each of the experimental groups. A significant difference is evident only for group 4 (***p < 0.0021). Bottom row: peak amplitude and area of the initial dip following condensed whisker stimulation protocol at baseline vs. 24 h for each of the experimental groups. Graph shows a significant difference only for group 4 at baseline vs. 24 h for both peak and area (***p < 0.0021 and **p < 0.03). There was no significant difference between the baseline values of all groups for all quantified parameters (*p* > 0.1).

However, the difference between experimental groups 2 and 4 is seen at 24-h after the 4-Cin treatment. Only group 4 shows absence of WFR whereas group 2 shows full recovery of WFR (Figs. 5 and 6). These results were consistent for both condensed and sparse whisking protocols.

### Representative results for experimental groups

Figure 5 (upper half) shows for each experimental group, a representative case of a whisker functional representation (WFR) at baseline vs. 24 h following sparse stimulation protocol. For experimental group 4 no WFR was recovered 24 h after pMCAo. Conversely, for all the control groups (1–3) the WFR remains intact 24 h after the interventions. Figure 5 (lower half) shows for each experimental group a representative case of a whisker functional representation (WFR) at baseline vs. 24 h following condensed stimulation protocol. Again, for experimental group 4 no WFR was recovered 24 h after pMCAo. Conversely, for all the control groups (1–3) the WFR remains intact 24 h after the interventions.

### ISOI-WFR spatial analysis

Experimental results at baseline and 24 h for areal extent and peak amplitude as quantified for the initial dip and overshoot phases during application of the sparse protocol are shown in Fig. 6 upper and middle parts. Only group 4 shows a significant decrease in both the peak value and areal extent (denoted as ‘area’) of initial dip and overshoot. We further verified the complete elimination at 24 h by absence of imaged WFR even at the lowest quantification threshold. Baseline values for all the other groups do not show any significant difference. Results show no significant effect for vehicle (Dmso) application after pMCAo in group 3, or during normal conditions in group 1. Lactate transport inhibition in the sham pMCAo group 2 also did not show any significant change in the area size and peak value. Figure 6 (bottom) shows the same spatial parameters of the initial dip during application of the condensed whisking protocol. It shows a similar trend as in Fig. 6 upper and middle parts, where the loss of spatial parameters is only evident in group 4, and all the other groups show no significant effect at 24 h after the treatment.

### ISOI-WFR temporal analysis

Experimental results for temporal parameters of the timing analysis are shown in supplementary Figure S2. Like spatial analysis, only in group 4 there was a significant decrease in temporal parameters such as half-time ratio and peak time ratio of both initial dip and overshoot phases after 24 h. All the other groups did not show any significant difference, indicating no significant effect of the vehicle (Dmso) after pMCAo (group 3) and during normal conditions (group 1). Blocking lactate transport in sham rats also did not show any significant change at 24 h (Figure S2), and only a temporary inhibitory effect of the WFR during 4-Cin administration (Fig. 4). All the other timing parameters (Time durations, rising and falling rate, minimum to maximum ratio and area under the curve) measured for the experimental groups also show significant change only in the group where lactate shuttle is blocked after pMCAo (group 4). The non-significant change in all the parameters for all other groups (1–3) provided further proof of perseverance of functional response in presence of vehicle and in Sham animals.

### Blocked ANLS rats show cortical infarct

**Figure 7:**
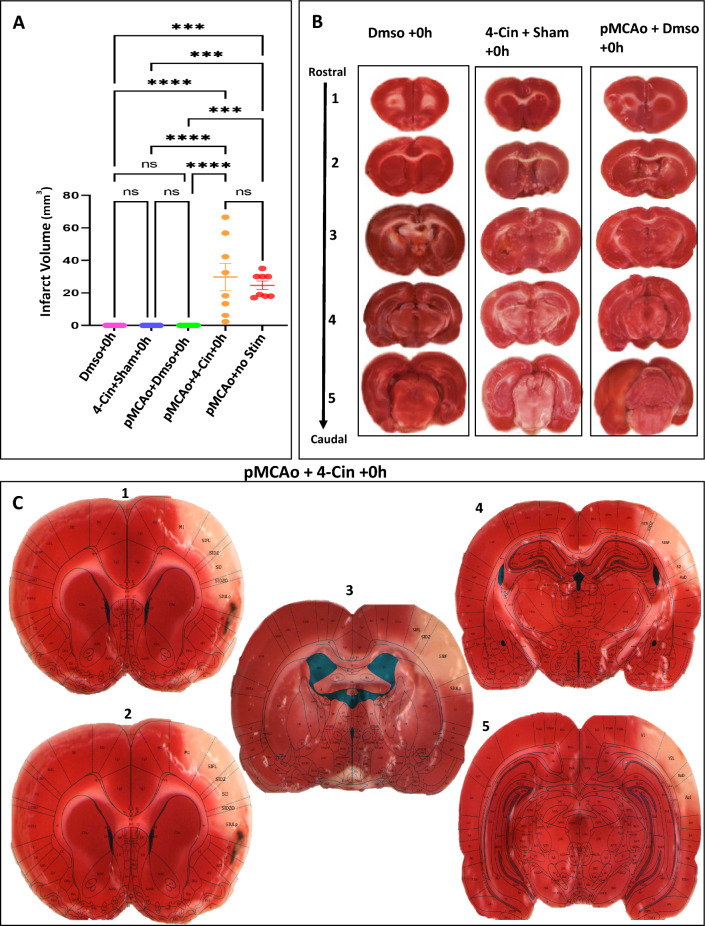
TTC quantification of infarct, or lack of, in all experimental groups. (**A**) Infarct volume from all the experiment groups (1–4). The infarct volumes for group (pMCAo + no-stim) are added as reference, taken from our previous study^5^. Group quantification (n = 8 in each group) of the infarct’s volume (****p < 0.0001). (**B**) Representative examples of TTC-staining for infarct revealed no damage and complete structural preservation in all other groups (1–3). (**C**) Brain slices show a TTC-stained representative case for group 4 in 2 mm slices with superimposition of TTC result on images from rat brain atlas (Paxinos and Watson^41^).

The infarct volume was calculated for all the experimental groups (1–4) in Fig. 7A. Another group with pMCAo and no + 0 h stimulation taken from our previous study is added as reference^5^. Brain slices from a representative selected animal for each of the group 1–4 are shown in Fig. 7B,C. The superimposition of Paxinos and Watson’s rat brain atlas according to the slices’ landmarks in Fig. 7C, show the resulting infarct following pMCAo in a case where the lactate transport is blocked over large cortical area (largest case of skull-dura-slits). The superimposition of TTC slices with the rat brain atlas shows infarct covering parts of primary motor cortex, primary somatosensory cortex, auditory cortex all the way up to parts of primary visual cortex, spanning almost the entire MCA territory^42^.

### Infarct volume is directly correlated to the size of slits

**Figure 8:**
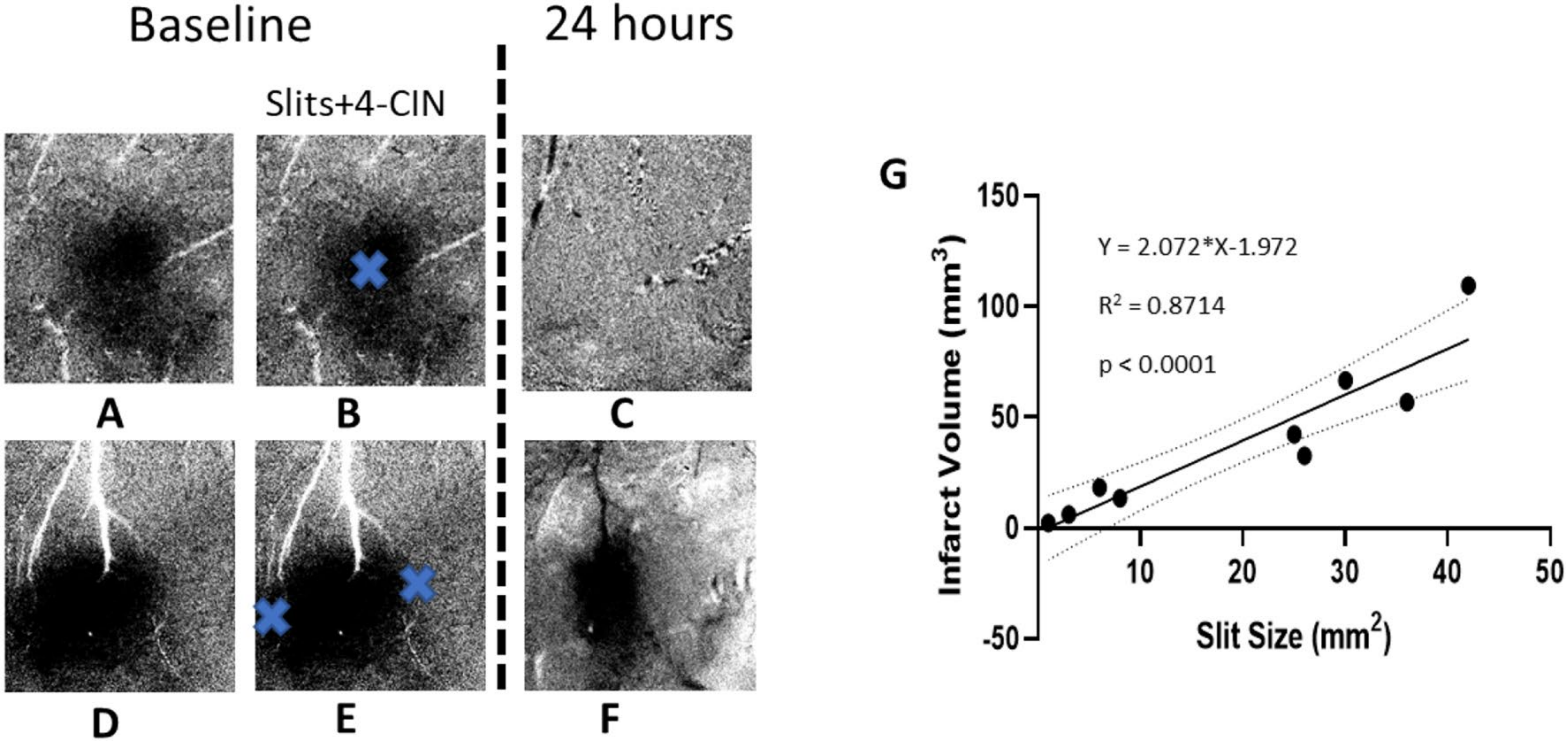
Dura slits’ spatial relationship to ISOI-WFR and infarct volume. The data from 2 rats are shown in (**A**–**F**). (**A**,**D**) The images show WFR imaged acquired using sparse protocol. Only initial dip is shown for two animals. (**B**,**E**) show the approximate location of the slits in dura and thinned skull as blue crosses. (**C**,**F**) WFR 24 h after pMCAo and 4-Cin treatment. Slit size and infarct size correlation: in C the WFR is absent and in F only the center remains. (**G**) shows linear regression of total slit(s) size and infarct. The infarct volume shows a strong linear correlation with the total slits size (R^2^ = 0.87, *p* < 0.0001). Black and white streaks are large surface blood vessels artifacts.

The location of infarct was always found directly under the slit as shown in Fig. 8A–F. One skull-dura slit eliminated the WFR directly under it (Fig. 8A–C), and two different locations around another WFR show infarct at two locations around the WFR, preserving function and structure of cortex not covered by dura slit (Fig. 8D,F). Therefore, functional, and structural preservation is directly correlated with the size of the slit created for drug diffusion to block the MCTs for lactate transport as shown by our results. For all the rats in group 4 (pMCAo + 4-Cin + 0 h), our results show a strong linear correlation between the size of slit to the volume of infarct as shown in the regression analysis (Fig. 8G).

## Discussion

In healthy rodents, astrocytic glycogen, a precursor of lactate, is the only endogenous fuel reserve for energy management during metabolically intensive or stressed conditions^43–45^. Astrocytic glycogen reserve is dynamically maintained through glycogenesis and glycogenolysis ^46,47^. Astrocytic glycogen is further metabolized during a metabolic demand of active neurons to generate lactate and therefore supplement such active neurons with an additional energy source to maintain functionality^15,27,28^. This process starts when evoked synaptic activity causes glutamate release at cortical synapses that in turn triggers glucose uptake in astrocytes and its metabolism through aerobic glycolysis resulting in lactate production, constituting one metabolic pathway of the astrocyte-neuron lactate shuttle (ANLS)^48,49^. During bursts of increased neuronal firing the glycogenolytic flux also increases and the astrocytic glycogenolytic pathway provides rapid lactate release to match intensive energy requirement^29,50^. The lactate produced via glycolysis and glycogenolysis in astrocytes is released to the extracellular space through monocarboxylate transporters (MCTs) to be consumed by an oxidative site, primarily neurons, constituting the pathway of astrocyte-neuron lactate shuttle (ANLS)^51^. The role of lactate produced by astrocytes in response to sensory stimulation within the critical 2 h time window of protection had not been tested before. We therefore hypothesized that the ANLS in response to sensory stimulation of neurons in the ischemic area could be the complementary mechanism to collateral blood flow in the process of protection by whisker stimulation in rat model of pMCAo. To test our hypothesis, we used quantification of ISOI following two stimulation protocols, in pharmacologically treated groups of rats to study in real time the role of astrocytic lactate in vivo.

Solving two key issues was instrumental for the success of the pharmacological applications in this study: 1) the novel development of the aligned skull-dura slits, and 2) the choice of the appropriate concentrations of the vehicle Dmso and MCT blocker 4-Cin.

1) For drug administration (Figs. 2 and 3) we created aligned skull-dura slits at or around the site of activity, avoiding major pial vascular network. This preparation has several advantages: a) Controlled drug diffusion over time only at the slits region, b) Being superior to an intracerebral injection by avoiding damage to cortex, c) No mechanical disturbances and drug pocket in the cortical milieu due to pressure injection, d) This procedure does not limit the drug volume as is the case in injections, e) keeping the thinned skull preparation with small sized slits, as small as 1 mm, eliminates cortical herniation and motion artifacts produced by heartbeat and respiration, and finally f) the size of the slits are linearly correlated with the cortical volume that is affected by the pharmacological intervention (Fig. 8G).

2) The use of Dmso has been reported to be potentially toxic above a certain dosage, therefore for this study it was important to use a concentration which was not toxic to the cortical milieu^52,53^ as verified by the ISOI. Indeed, our results in control groups 1(Dmso without pMCAo) and 3(Dmso + pMCAo) demonstrated that no spatial or temporal changes were detected for the WFR, verifying that the concentration of Dmso used for our study had no toxic effects. The concentration of 4-Cin used in this study has been in the middle range of what has been previously used for MCT inhibition^54–56^. This concentration was chosen by considering that the dynamic diffusion through the cortical extracellular space does not minimize the efficacy of the drug, and conversely that it does not exceed a concentration that could have any spatial or temporal effect on the WFR during normal condition, as shown in control group 2 (sham pMCAo + 4-Cin + 0 h).

We used ISOI in combination with pharmacological manipulation of lactate transporters to reveal the role of ANLS in sensory-based protection following pMCAo by quantification of the WFR over time. Previously, we have shown that despite pMCAo, rats show cortical structural, functional, and behavioral protection following whisker stimulation within 2 h window after pMCAo^1,2,4^. By pharmacologically inhibiting MCTs using 4-Cin, lactate transport is blocked between astrocytes and neurons resulting in both immediate and 24 h abolishment of the WFR in addition to causing an infarct as seen in postmortem histology. These functional and structural results highlight the pivotal role of lactate shuttle in the neuroprotection process following pMCAo in rats. Further proof of ANLS support in neuroprotection of our pMCAo model is evident by our finding that the volume of infarct is directly proportional to the size of the dura slits or the volume of cortical region that had ANLS inhibited, as shown in Fig. 8.

An important finding of our study is that we have demonstrated, blocking the ANLS transport in normal (sham, group 2) conditions during the application of 4-Cin had also strong obliterating effects on the imaged WFR, evidenced by the complete disappearance of all three distinct WFR phases (Fig. 4). Corroborating these results, previous studies reported that blocking lactate transport by downregulation of MCT transporters (MCT2 or MCT4) abolishes cortical evoked BOLD response to whisker stimulation (equivalent the ISOI overshoot phase) as measured by fMRI and by nuclear magnetic resonance spectroscopy^57–60^.

We were able to generalize our findings using two very different types of whisker stimulation protocols. A sparse protocol, which was used in all our previous protection studies, was applied in this study with the advantage of being able to quantify two out of the three phases (initial dip and overshoot) that can also be imaged by high-powered BOLD-fMRI following sensory stimulation. The condensed protocol shows only one phase, but its advantage is in its similarity to whisker stimulation parameters in naturally whisking rats. Our results show that the spatial and temporal WFR is preserved in all the different conditions of interventions and drugs used (groups 1–3) for both whisker stimulation protocols and consequently demonstrate that ANLS-based protection is successful regardless of these very different stimulation protocols.

After years of controversy regarding the source of energy for active neurons, it was recently demonstrated that in the healthy brain the source of energy to active neurons critically depends on the level of neuronal activity. At low neuronal activity levels neurons utilize blood glucose whereas higher level of neuronal activity induces a switch to lactate use obtained through the ANLS, rather than glucose^15^. In the ischemic cortex, whisker stimulation, as applied in the current study, provides a strong activation of the ischemic cortex. Our results indeed demonstrate the activation of the ANLS in the ischemic cortex following neuronal activation, therefore suggesting that similar rules regarding the source of energy to active neurons could also apply following pMCAo. However, our results could not differentiate which type of ANLS was activated as the source of lactate, i.e., glycogenolysis vs. aerobic glycolysis in astrocytes, and further studies are needed to disambiguate the ANLS origin. In addition, our study has only demonstrated that the ANLS is a necessary part of the protection by whisker stimulation but didn’t prove a direct causal link between whisker stimulation and the ANLS, which necessitates further experiments to establish such link. With blocked ANLS, neurons could still take up glucose through glucose transporters from the collateral blood flow potentially providing lactate for energy consumption. This possibility is unlikely for three reasons. First, using DOCT, a technique that allows for quantification of blood flow and flux, the sensory-based evoked collateral blood flow following pMCAo, was found to be only about 10% of the baseline levels^9^ and therefore such low flow/flux levels is not an optimal source for glucose. Second, hypoxic environment favors glycogenolysis^21^. Third, the elimination of the WFR and presence of infarct in group 4 (pMCAo + 4-Cin + 0 h) are clear indications of cortical dependence on ANLS and therefore rule out direct neuronal glucose use after pMCAo.

Some researchers have also suggested that glycogen content during basal condition in the healthy rat brain is in a very minimal amount (10–12 micromole/g), which can only help in sustaining the tissue for only few minutes after the ischemic insult^61^. However, our previous and current results do not support the case of ‘few minutes of support’ following pMCAo as we have previously shown that following pMCAo the cortex is still protected for at least 2 h even without any sensory stimulation^1,2^. The current study shows different results from blocking of the ANLS in experimental groups 4 (pMCAo + 4-Cin + 0 h) and 2 (sham pMCAo + 4-Cin + 0 h) suggesting that the ANLS is supporting neurons beyond few minutes, otherwise its inhibition of the 2-h protective time-window should not have blocked any neuroprotective effects later, contrary to our results in group 4. Notably, glycogen, in addition to being a precursor of lactate, is also a precursor of the cortical neurotransmitter glutamate required for neuronal stimulation^46^. In response to the increase in cytosolic Na^+^ astrocytes take-up glutamate and recycle it through glutamate-glutamine cycle and activate glycolysis/glycogenolysis resulting in ANLS to make up for neuronal energy demand^62^. As neuronal activation due to sensory stimulation increases, more glutamate is released, and consequently more glycogen is used to support the active neurons^20^.

Following pMCAo, another potential source of astrocytic activation (to trigger ANLS that supports ischemic brain) could be related to changes in subthreshold neuronal cortical activity within the ischemic cortex. Our previous results using microelectrode array recording in the same rat model have demonstrated that within few minutes following pMCAo, there is a remarkable widespread buildup of tight spatiotemporal synchronization of spontaneous neuronal activity local field potentials (LFPs) over the entire cortical depth and the entire spatial extent of the occluded MCA territory, without any parallel change in sensory evoked LFPs and spikes as compared to pre-pMCAo baseline. Such LFP synchronization following pMCAo is the result of underlying synchronous bursts of low frequencies oscillations^23^. The continuous buildup of such synchronization over time results in an infarct, unless whisker stimulation is delivered to the ischemic cortex during the 2 h protective window resulting in desynchronization of the correlated LFPs and protection from impending infarct as verified by postmortem histology^24^. The widespread synchrony of low frequency bursts underlying the LFP synchrony buildup following pMCAo could also contribute to high energy demand to support this synchronous burst activity, which in turn could therefore also result in increase in demand for lactate. These findings, together with the contributions of the collaterals support system and the ANLS support system demonstrate that protection by sensory stimulation following pMCAo is a multi-dimensional integrated activity that involves neurons, astrocytes and blood vessels, all members of the NGV-unit with astrocytes being the key regulator of neurometabolic and neurovascular coupling^63^.

A major finding of this study is that ANLS is a pivotal neuroprotective component along with collateral blood flow during the hyperacute state following pMCAo. Most of the relevant preclinical and clinical work to date has focused on administrating exogenous lactate after ischemia, which enhances the neuroprotection by reducing the infarct lesion of ischemic region^19,64–72^. Our findings show that sensory-based stimulation of neurons and astrocytes in ischemic area allows for protection via ANLS without the need of external lactate administration, demonstrating the ability of the cortex to sustain itself following pMCAo, if neuronal stimulation is delivered within the critical time window for protection.

## Conclusion and outlook

In conclusion the results of this study are consistent with previous studies that suggest that lactate shuttle is important for neuronal functional preservation especially in a pathological condition like ischemia and highlights the important component of neuronal activation that has been mostly overlooked in previous studies. Astrocytic lactate shuttle in response to neuronal activation together with collateral blood flow and LFP desynchronization could all potentially participate in the protection of the ischemic cortex in our model of pMCAo. In the future, we believe that our pMCAo stroke model coupled with sensory-based neuroprotection in rats, and with better understanding of why such protection fails in mice^73^ and in hypertensive rats^74^, could further expand ways to protect the ischemic cortex from impending infarct.

### Supplementary Information

Supplementary Information.

## Data Availability

The dataset generated from raw images is available on request from corresponding author.
