# Supplementary material for: Astrocyte-neuron lactate shuttle plays a pivotal role in sensory-based neuroprotection in a rat model of permanent middle cerebral artery occlusion
Source: Sci Rep. 2023 Aug 7;13:12799. doi: 10.1038/s41598-023-39574-9 (PMC10406860; doi:10.1038/s41598-023-39574-9)
Supplement: Supplementary file 1 — Supplementary Information. [file 41598_2023_39574_MOESM1_ESM.pdf]

Astrocyte-neuron lactate shuttle plays a pivotal role in sensory-based neuroprotection in a rat model of permanent middle cerebral artery occlusion.

Mehwish S. Bhatti[1*], and Ron D. Frostig[1,2,3*]

[1] Department of Neurobiology and Behavior, School of Biological Sciences, University of California, Irvine, Irvine, CA, USA
[2] Department of Biomedical Engineering, School of Engineering, University of California, Irvine, Irvine, CA, USA
[3] Center for Neurobiology of Learning and Memory, University of California, Irvine, Irvine, CA, USA, *rfrostig@uci.edu, *mehwishb@uci.edu

Supplementary Materials:

Materials and Methods

Temporal Analysis:

The timing parameters for initial dip and overshoot were obtained as shown in the Figure S1. For temporal analysis of WFR, the two phases were selected by bounding boxes (using our customized-interactive GUI) for initial dip and overshoot (sparse protocol). The obtained parameters are:

i) Area duration: entire duration of evoked activity, T1 ii) Area half-duration: time duration of evoked activity at activity-half-width, T2 iii) Peak-max time: latency to peak value, T3. iv) Half-time ratio (HTr): ratio of the time duration at activity-half-width to total duration of evoked activity (T2/T1 X 100). v) Peak-time ratio (PTr): ratio of the peak-max time to entire duration of the evoked activity (T3/T1 X 100). The ratio of minimum value to maximum value for the temporal profile. A1-A10 are various regions calculated above and below the ISOI-WFR-temporal profile of activity (initial dip/overshoot) inside the bounding box selected for the activity as shown in Figure S1B. These area regions are used to calculate Falling rate (FR), Rising rate (RR) and area under the curve (AUC) as:

Falling rate = 100 X (A6 + A8) / (A6+A7+A8+A9+A10)

Falling Rate = 100 X (A3+A5) / (A1+A2+A3+A4+A5)

AUC = A3 + A5 + A6 + A8

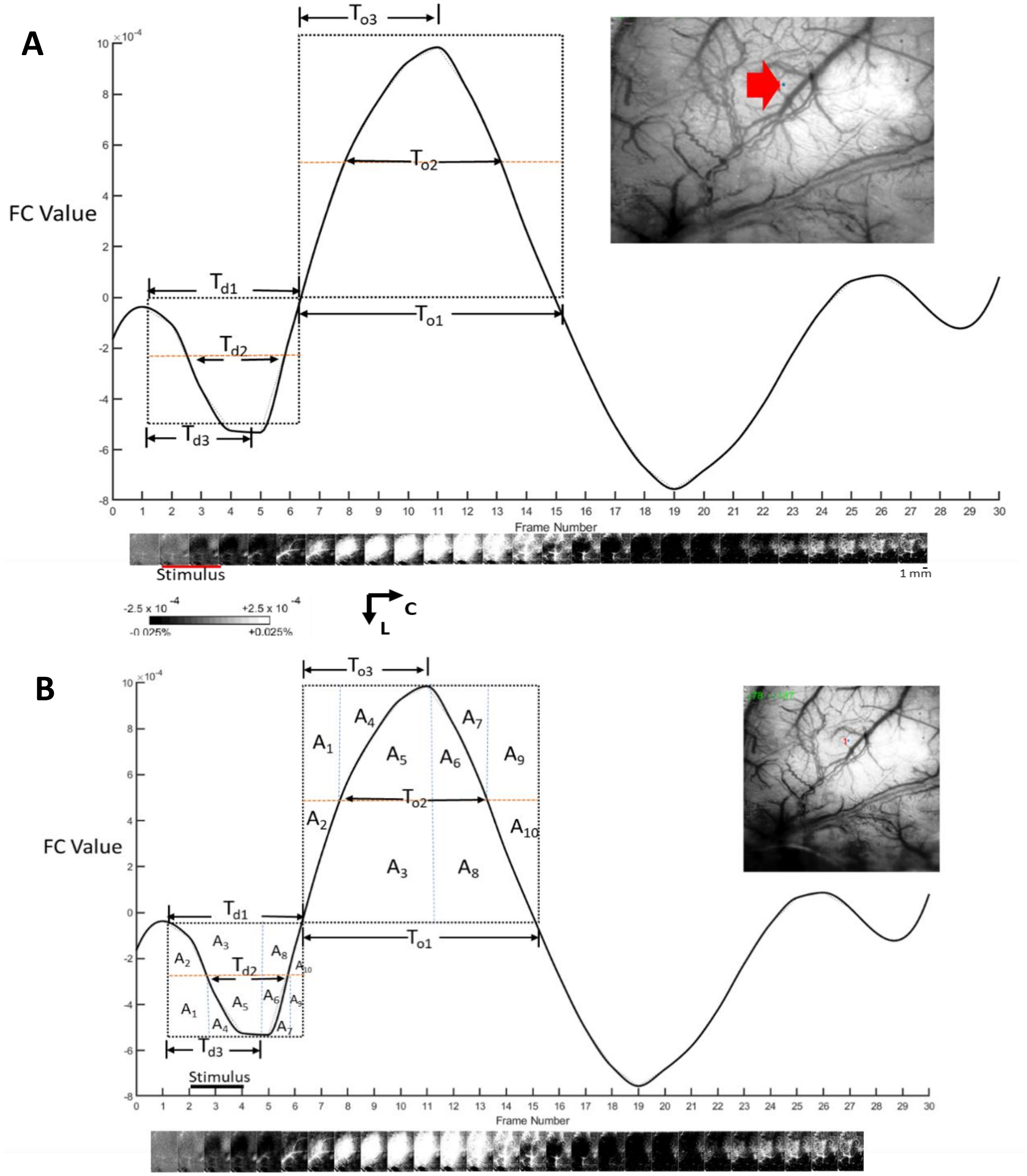

Figure S1. Temporal parameters of a WFR. The image at the top shows the selected pixel (in blue, marked by red arrow) and the graph shows the temporal profile of this pixel. Using bounding boxes (as shown by dotted boxes), two regions of interest are selected containing initial dip and overshoot. For the initial dip the bounding box covers the area from the start of baseline value to a minimum value and then back to the baseline value. For overshoot the bounding box covers the area from baseline to maximum value and then back to baseline. A) The time durations are marked as Td1-Td3 for initial dip and To1-To3 for overshoot. Td1/To1 are the total durations, Td2/To2 are the half-time, and Td3/Tx3 are the time to peak value of activity (minima for initial dip and maxima for overshoot). The sequence of images at the bottom shows the ISOI-WFR over time. Linear gray scale bar indicates intrinsic signal strength, C and L denotes caudal and lateral respectively. Each frame is ~7 mm x 7 mm. Black and white streaks are large surface blood vessels. B) The area regions (labelled: A1-A10) calculated for both initial dip and overshoot are measured inside the bounding box selected for the activity.

Results – Temporal Analyis

Parameter	Initial Dip	Overshoot
**T1**	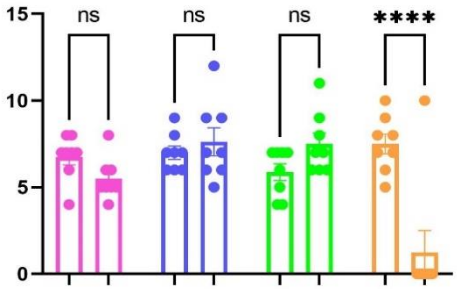	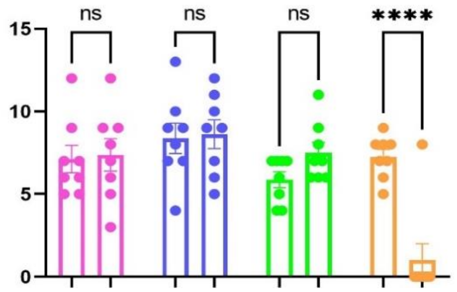
**T2**	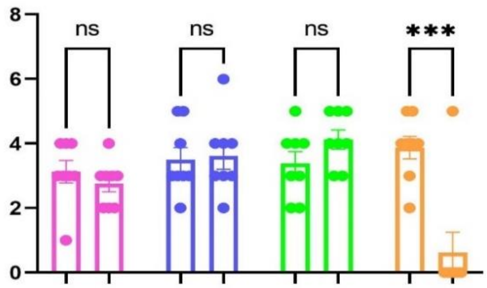	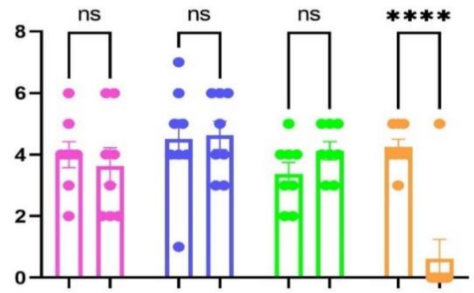
**T3**	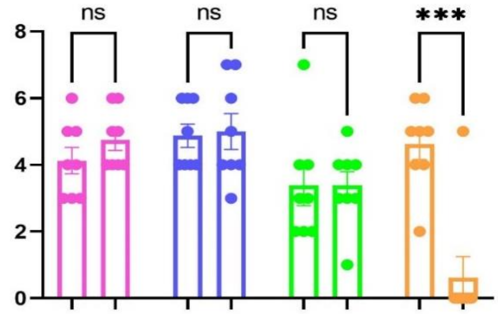	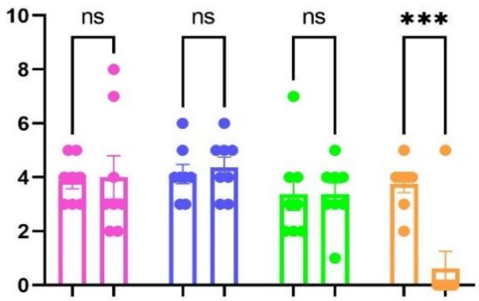
**HTr**	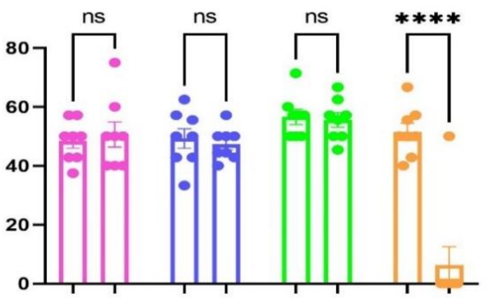	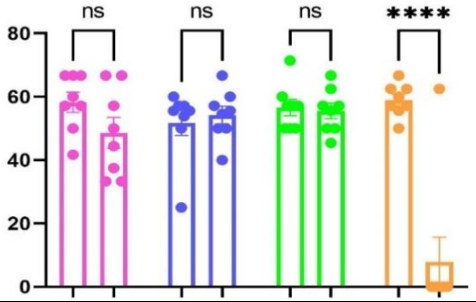

PTr

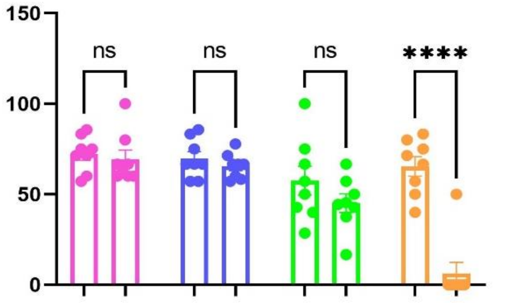

150
100
50
0
ns ns ns ****

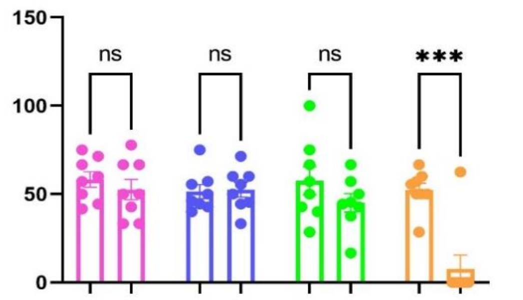

150
100
50
0
ns ns ns ***

FR

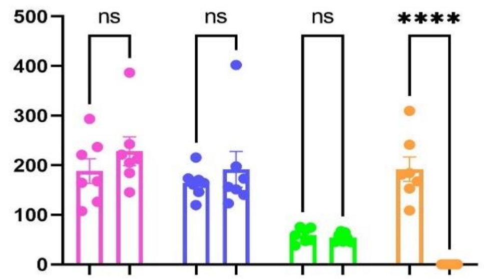

500
400
300
200
100
0
ns ns ns ****

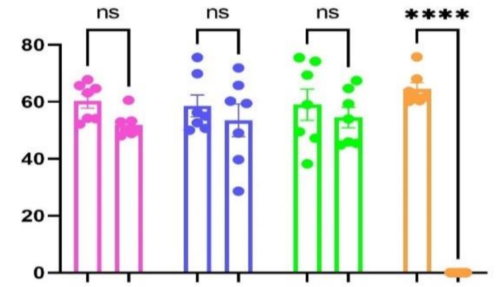

80
60
40
20
0
ns ns ns ****

RR

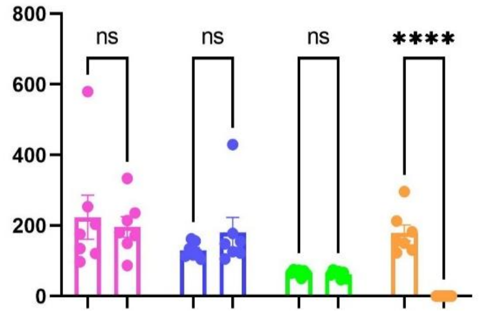

800
600
400
200
0
ns ns ns ****

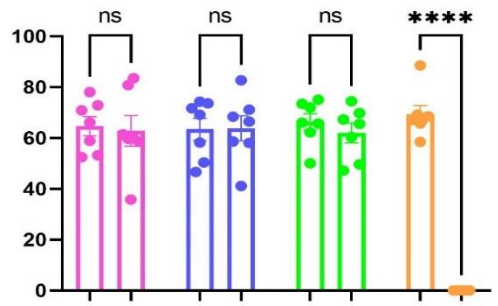

100
80
60
40
20
0
ns ns ns ****

AUC

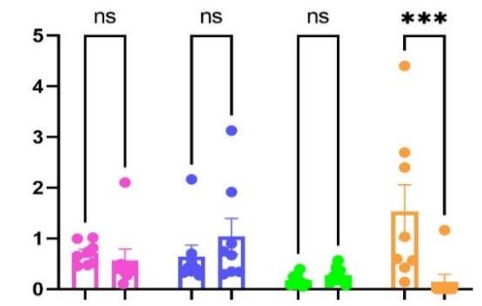

5
4
3
2
1
0
ns ns ns ***

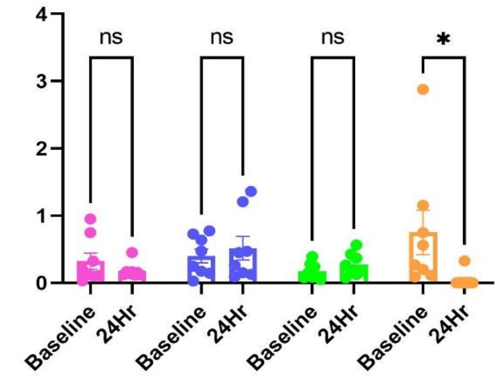

4
3
2
1
0
ns ns ns *
Baseline 24Hr Baseline 24Hr Baseline 24Hr Baseline 24Hr

**Min-Max Ratio**	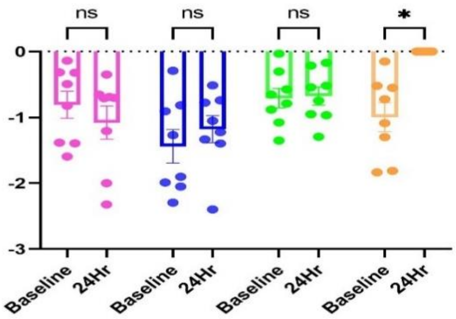
**1: Dmso + 0-hr**<br>**2: 4-Cin + Sham + 0-hr Stim**	**3: pMCAo + Dmso + 0-hr Stim**<br>**4: pMCAo + 4-Cin + 0-hr Stim**

*Figure S2. The temporal quantification of ISOI-WFR phases during sparse stimulation protocol. The timing parameters for Initial Dip and Overshoot are shown for all the experimental groups P1-P4. All the timing parameters for both initial dip and overshoot show a significant decrease for group P1(*p<0.05, ***p<0.0021 and ****p<0.0001). Control groups P2-P4 at baseline and 24 hours do not show any significant change. There was no significant difference between the baseline values of all groups for all quantified parameters (p>0.1).*

The timing parameters measured for all the groups 1-4 also show significant change only in the group where lactate shuttle is blocked after pMCAo (Group 4), Figure S2. The non-significant change in all the parameters for groups 1-3 provided further proof of perseverance of functional response in presence of vehicle and in Sham animals.
